# A porcine model of chronic hepatitis E virus (HEV) infection identifies male reproductive glands as sites of viral persistence

**DOI:** 10.1080/21505594.2026.2707701

**Published:** 2026-07-22

**Authors:** Kush K. Yadav, Patricia A. Boley, Thamonpan Laocharoensuk, Saroj Khatiwada, Carolyn M. Lee, Menuka Bhandari, Juliette Hanson, Scott P. Kenney

**Affiliations:** a Center for Food Animal Health, Department of Animal Sciences, The Ohio State University, Wooster, OH, USA; b Department of Veterinary Preventive Medicine, The Ohio State University, College of Veterinary Medicine, Columbus, OH, USA; c CFAES Research and Analytical Service Cores, The Ohio State University, Wooster, OH, USA; d National Bio and Agro-Defense Facility (NBAF), Manhattan, KS, USA

**Keywords:** Sperm, male, reproductive, hepatitis E, immunosuppressed, pig

## Abstract

Hepatitis E virus (HEV) is an expanding zoonotic viral disease threat. Although HEV causes acute viral hepatitis, it is increasingly being recognized as a systemic pathogen with detection and damage in extrahepatic tissues. The presence of HEV RNA in the semen of chronically infected human patients in the absence of viremia and fecal shedding and presence of HEV in the sperm head underscores the need to understand the interaction of HEV within the male reproduction system. The role of male accessory glands in the dissemination and persistence of HEV infection has not been studied. Using an immunosuppressed pig model for chronic HEV infection, we demonstrate infectious HEV in mature sperm cells, altering the sperm motility and morphology. HEV isolated from sperm cells remained infectious in human hepatoma cells. Spermatic fluid contained lower virus titers than the sperm cells from chronically infected pigs, highlighting that the sperm cells themselves can associate with the virus. Evaluation of the male accessory glands demonstrated viral replication, infiltration of CD45 leukocytes, and apoptosis associated with HEV infection. A decrease in serum testosterone levels was evident in the HEV-infected pigs. Even though a lower viral RNA titer was seen in serum and feces of chronically infected, immunosuppressed, and ribavirin-treated pigs, high viral RNA and infectious particles in sperm is a concern. Our findings necessitate further studies defining the mechanism of sperm cell invasion by HEV and the risk of sexual transmission of HEV during both acute and chronic phases of infection.

## HIGHLIGHTS

- Hepatitis E virus is associated with the sperm head in immunosuppressed ribavirin-treated HEV-infected pigs.
- We demonstrate that the male accessory sex glands may have a role in the persistence of HEV infection during chronic infections.
- Our study further highlights the importance of the testis, as an immune-privileged site, in the maintenance of chronic HEV infection.

## Introduction

Hepatitis E virus (HEV) is a major challenge for medical and scientific communities. Among the many issues complicating HEV disease are increasingly complicated extrahepatic manifestations [1]. These extrahepatic manifestations are most frequently encountered with immunosuppressed patients [2–4]. HEV in tissues beyond the liver and its association with the disease have shifted the study of hepevirus–host interactions, expanding the need to examine sites outside of the liver to fully understand HEV pathogenesis.

A majority of the chronic cases are due to genotype (gt) 3 and gt4 *Paslahepevirus balayani* strains [2,5]. Studies show 10% of chronically infected patients have higher chances of developing cirrhosis within 2–5 y [6]. The first step in treating chronic HEV infection in solid organ transplant patients is to reduce the immunosuppressive drugs followed by the administration of ribavirin (RV) [7,8]. It has been documented that the achieved sustained virological response (SVR) rate is 91%, 76%, 67%, and 63% in liver, kidney, lung, and heart transplant patients, respectively [7]. Additionally, no response was documented in 6% of chronically infected patients, and relapse has been shown in 18% of chronically infected patients [6,7]. The detection of HEV in the ejaculate of seven out of nine chronically infected men highlights the need to understand the reproductive tissue’s role in the maintenance of HEV infection [9,10]. The lower SVR rate and relapse in chronically infected patients raises concerns about a potential immune-privileged tissue reservoir, such as testis role in the persistence or relapse of chronic HEV infection.

Recently, HEV RNA has been detected in the semen of seven chronically infected human patients [9] and in human semen samples where the presence of HEV RNA correlation with infertility is still debatable [11,12]. Interestingly, in one chronically infected patient, viremia and fecal virus shedding had subsided with only semen demonstrating HEV RNA [9]. The seminal HEV RNA concentration was reported to be 100-fold higher compared to serum viremia among the five other chronically infected patients [10]. Previously, we defined HEV association with the sperm head altering sperm quality in pigs [13]. We found infectious virus associated with sperm cells when tested *in vitro*. We demonstrated higher HEV titers from sperm cell fractions than in the seminal fluid using a natural HEV host, pigs [13]. Our initial findings demonstrated that the pig could be a good reproductive model to assess HEV effects in male reproductive disorders [13].

Human male reproductive accessory glands, such as the prostate gland, seminal vesicle, and Cowper’s gland, are also found in pigs allowing the pig to be used as a model to study the male accessory glands [14]. Pigs, as a natural host for HEV, have served as a model organism for pathogenesis studies [15]. Interestingly, HEV effects in the male reproductive system have been correlated to infertility and testicular damage using animal models, such as rhesus macaques [11], mice [16], and Mongolian gerbils [17]. However, the role of male accessory glands in the contribution of HEV in the sperm cells and seminal fluids has not been studied. We hypothesize that HEV has the potential to replicate in the male accessory glands that contribute to the HEV in the semen. Hence, the objective of this study is to understand the role of male accessory glands in HEV replication and persistence.

To mimic the immunosuppressed chronically infected human patient, we utilized an established chronic HEV pig model to understand the effect of HEV in sperm quality and morphology [15]. We further studied the effect of HEV on blood–testis barrier (BTB) destruction, infiltration of leukocytes into the testis, testosterone concentration, and contribution of male accessory glands in the persistence of HEV during chronic HEV infection. In addition, we compared the immunosuppressed pig model data to immunocompetent pigs to understand the overall infection pattern in a comparative fashion. Answers to these questions are important to understand the tissue reservoirs of HEV in the male reproductive system and the ability of the virus to transmit sexually between partners.

## Materials and methods

### Ethics statement

All animal experiments in this study were approved by The Ohio State University Institutional Animal Care and Use Committee (IACUC 2020A00000068) and virus studies were approved by the Ohio State Institutional Biosafety Committee (IBC 2016R00000082).

This study was conducted and reported in accordance with the ARRIVE (Animal Research: Reporting of *In Vivo* Experiments) guidelines (ARRIVE 2.0) to ensure transparency, reproducibility, and ethical rigor in animal research.

### Animals, immunosuppressive drugs, and virus

All pigs (Cross of white Landrace and Duroc) used in the study were male, 6-week-old conventional pigs. Pigs were randomly assigned to treatment groups based upon offloading order from transport trailer (Table 1). Pigs were floor-housed in climate-controlled biosafety level 2 rooms and fed the same quantity of feed twice daily. Pigs were acclimatized to the environment for 10 d before the start of the experiment and were screened for HEV fecal shedding (negative) and HEV antibody titers (negative). Prior to experimental infection, animals were tested and confirmed negative for rotavirus and mycoplasma infections, which are considered sporadic in the region. Treatments were given at the same times by trained handlers. A total of eight pigs (n1 = 4 and n2 = 4) were administered immunosuppressive (IS) drugs (cyclosporine: 10 mg/kg/d, prednisolone: 4 mg/kg/d, and azathioprine: 2 mg/kg/d) following the established regimen from the Meng laboratory [15]. These two groups (n1 and n2) were also administered ribavirin (RV): 80 mg/d. Male conventional pigs (n1 = 4) in the US-2 HEV-infected group were anesthetized with TKX (telazol, ketamine, and xylazine) at 0.02–0.04 mL/kg and infected via ear-vein inoculation with (2 × 10^8^) viral RNA copies (log_10_/mL) of gastrointestinally derived genotype 3 US-2 (human) HEV (GenBank accession no. AF060669) [18]. The remaining four pigs (n2 = 4) only continued with the immunosuppressive drugs and ribavirin throughout the study. Four pigs (n3 = 4) were used as a negative control in the study and the other four (n4 = 4) were only infected with US-2 HEV.

**Table 1.** Experimental groups in the study.

Group 1	Immunosuppressive (IS) drugs + Ribavirin + US-2 HEV
Group 2	Immunosuppressive (IS) drugs + ribavirin
Group 3	US-2 HEV
Group 4	Negative control

These pig samples were utilized from a previous study [13] to prevent the unnecessary and duplicitous use of animals for this study. This intravenous route of infection mimics what might occur in the case of human transfusion–acquired HEV infection. Sample size was based on the differences noted in our previous study with 4 pigs/group. The animal numbers were sufficient to answer the study question following the 3 R (replacement, reduction, and refinement) rules.

### Collection of semen from US-2 HEV-inoculated pigs

Testes were collected from pigs at 84 d postinoculation (dpi) (age of pigs = 126 d old). Euthanization was done with pentobarbitol (1 mL/10 lbs) directly injected to the heart, followed by blood drainage by hanging the pig upside down. The testis was extracted during necropsy by a veterinarian and stored in a cooled box. Semen from the epididymis was harvested by aspiration and flushing [19]. The procedure was repeated for both testes from the same animal, and the extracted semen was pooled.

### Examination of semen parameters

Semen samples were examined within 15 min after collection following the 2010 World Health Organization laboratory manual for the examination of human semen [20]. Motility and morphology of sperm cells between the groups were studied. In brief, 200 spermatozoa per replicate were examined at 200× magnification. Motility in a sperm cell was studied using three categories: progressive (PR), nonprogressive (NP), and immobility (IM). PR refers to a percentage of a sperm’s progressive motility (moving actively, either linearly or in a circle, regardless of speed). NP refers to nonprogressive motility (all other patterns of motility with absent progression). IM refers to complete immobility.

### Sperm cell separation from the semen

Semen is a mixture of seminal plasma fluid and sperm cells [21]. Sperm cells were separated using phosphate-buffered saline (PBS) in a ratio of 1:10 (volume/volume) and gently mixed to remove the sperm cell clumps. Supernatant was collected and stored at −80°C post centrifugation at 1500 rpm for 10 min. To remove any remnants of seminal plasma, the sperm cell pellet was washed once with 900 µL of PBS, centrifuged, and the supernatant was discarded. The remaining pellet was carefully resuspended with another 900 µL of PBS [22].

### Immunofluorescence staining of sperm cells

To identify HEV antigen in sperm cells, 15 µL of the sperm suspension was added in duplicate to Superfrost Plus Slides (Fisher) dried, and fixed with paraformaldehyde solution (4% in PBS) at 4°C for 5 min, followed by two rounds of washing in PBS for 5 min at room temperature. For the detection of HEV antigens, 1:200 rabbit anti-HEV open reading frame 2 (ORF2, encodes structural capsid protein of HEV) polyclonal antibody (Cocalico Biologicals from in-house prepared ORF2 protein lacking the first 111 amino acids produced in *Escherichia coli*, denatured, and polyacrylamide gel purified) was added to each slide. The slides were incubated for 45 min at room temperature followed by three washings in PBS for 5 min each. Goat antirabbit IgG H&L (Alexa Fluor 594; Abcam) (1:500) was used as the secondary antibody, and the slides were incubated for 30 min at room temperature. 30-µL of DAPI (4’,6-diamidino-2-phenylindole; 300 nM, Fisher) was added as a nuclear counterstain for fluorescence microscopy. Slides were imaged using a Keyence microscope at 40× magnification. A sample was considered positive if at least one cell with clear sperm-like morphology contained red fluorescence. All specimens were examined in duplicate.

### Flow cytometry analyses

Four hundred microliters of the sperm cell suspension was used for flow cytometry quantification of HEV-positive cells. Fixed cells were probed with a primary antibody – rabbit anti-ORF2 HEV diluted 1:200 in PBS for 30 min at 37°C. Cells were then washed twice with PBS and incubated with a secondary antibody – goat antirabbit phycoerythrin (Life Technologies) diluted 1:500 in PBS for 30 min at 37°C. Cells were then washed and resuspended in 200 µL of PBS. Fluorescence was analyzed for 100,000 events using a flow cytometer (BD Accuri C6 Plus). Histogram plots were compared between the infected and mock-infected sperm cells.

### Infectivity assay

Four hundred and fifty microliters of the sperm cell suspension (~4 × 10^6^ viral RNA copies) was used to assess the infectiousness of HEV derived from sperm cells. Human liver cells (Huh7 S10-3) were used for the study [23]. Three repeated freeze and thaw cycles were done with the sperm cell suspension. Cell lysates, including both the cell debris and virus, were subjected to high-speed centrifugation (10,000 × *g* for 5 min) which separates the cell debris. The collected supernatant was used to inoculate Huh7 S10-3 liver cells. Eight hours postinoculation, the cell culture media was removed, and fresh media was added. Forty-eight hours postinoculation, cells were passed 1:3 and 72 h later were fixed for immunofluorescence assays.

### Indirect immunofluorescence

At 5 dpi, human liver cells (Huh7 S10-3) were fixed with 4% paraformaldehyde and permeabilized with PBS plus 0.5% Tween 20 (PBST). Five percent nonfat dried milk (Sigma-Aldrich) in PBST was used to block nonspecific antibody binding. Immunostaining of HEV ORF2 capsid protein was performed using 1:200 rabbit anti-truncated ORF2 HEV. A fluorescently labeled goat anti-rabbit IgG H&L secondary antibody (Alexa Fluor 594; Abcam), 1:500, was used. DAPI (Fisher) was used to stain the nucleus. Images were optimized for intensity enhancement using Coreldraw 2019 applied to all images equally.

### Accessory glands collection

Accessory glands were collected with the help of a certified veterinarian. Briefly, a piece of each accessory gland (Cowper’s gland, prostate gland, and seminal vesicles) was collected in RNA later solution (Invitrogen) for RNA extraction. A piece of each gland was also collected in 10% formalin. Formalin was replaced by fresh 10% formalin after 24 h.

### RNA extraction and RT-qPCR

RNA was extracted using TRIzol reagent (Invitrogen). Extracted RNA from serum, rectal swabs, sperm cells, and seminal fluid was quantitated using reverse transcriptase quantitative polymerase chain reaction (RT-qPCR). Primers US-2 HEV F, 5’-GGTGGTTTCTGGGGTGAC-3’ and US-2 HEV R, 5’-AGGGGTTGGTTGGATGAA-3’, and probe 5’-FAM-TGATTCTCAGCCCTTCGC-Dabcyl-3’ were used for the detection of US-2 HEV. A 10-fold serial dilution of US-2 HEV RNA (10^7^–10^1^ copies) was used as a standard for quantification of the viral genome copy numbers.

### Immunohistochemistry (IHC)

Formalin-fixed tissue sections were tested by IHC for the detection of HEV, as previously described with slight modifications [24]. The rabbit anti-HEV-ORF2 antibody was used as the primary antibody and a horseradish peroxidase-conjugated antirabbit antibody (BioGeneX) was used for visualization as brown staining. Stained tissues were counterstained with hematoxylin. In addition, the tissues were also tested by IHC for the detection of CD45-expressing cells. Mouse antipig CD45 (Bio-RAD) was used as the primary antibody and a horseradish peroxidase-conjugated antimouse antibody (BioGeneX) was used for visualization as brown staining.

### Testosterone assay

Serum samples from pigs were tested for testosterone concentration on d 0, 14, 28, 42, 56, 70, and 84 postinoculation using a pig testosterone enzyme-linked immunosorbent assay (ELISA) kit (CUSABIO, CSB-E06796p). Briefly, 50 µL of the sample was added in a well, followed by 50 µL of HRP-conjugate and 50 µL of antibody. The samples were incubated for 1 h at 37°C after mixing. Later, the solution was aspirated, discarded, and wells were washed three times with the wash buffer. Then, 50 µL of substrate A and substrate B were added to each well, mixed, and the plate was incubated for 15 min at 37°C in the dark. The stop solution was added, and the absorbance was read at 450 nm using a microplate reader (Filtermax F5, Molecular Devices).

### Terminal deoxynucleotidyl transferase dUTP nick-end labeling (TUNEL) assay

Apoptotic cells in the testicles of pigs were detected by using One Step Apoptosis Assay Kit (Abcam, ab206386) according to manufacturer’s directions. Briefly, tissue sections were deparaffinized, hydrated, blocked with proteinase K for 15–30 min, and washed with PBS. After quenching with the 3% hydrogen peroxide, tissue specimens were covered with TdT equilibration buffer and incubated for 30 min followed by a labeling reaction using the TdT labeling reaction mix. The tissue specimens were then treated with DAB solution, followed by a Methyl Green counterstain solution.

### qPCR to detect the apoptosis gene expression

A cDNA synthesis kit (iScript, Bio-Rad, Hercules, CA) was used to synthesize cDNA using total RNA extracted from tissues. The number of viral RNA copies was calculated and compared to the number of copies of the standard control. Primers used: GAPDH F: ACCCAGAAGACTGTGGATGG, GAPDH R: TCAGCTCAGGGATGACCTTG, BAX F: GACGGCAACTTCAACTGGG, BAX R: GCAGCCGATCTCGAAGGAA, Caspase 3 F: TCTAAGCCATGGTGAAGAAGGAAAAA, Caspase 3 R: GGGTTTGCCAGTTAGAGTTCTACAG. The generation of oligonucleotide dimers for each primer pair was assessed using Power Up SYBR® Green PCR MasterMix (Thermofisher) with melting curve analysis, according to the manufacturer’s instructions. One to 4 µL of cDNA sample was assayed per reaction. Each reaction consisted of 1 cycle of 50°C for 2 min, 1 cycle of 95°C for 2 min, followed by 45 cycles of 95°C for 15 s and 60°C for 1 min.

### Calculations of reaction efficiency and fold change of gene expression

The efficiency of each gene amplification was calculated by plotting the average Ct (*y*-axis) against the logarithm of the input amount of RNA/µL cDNA (*x*-axis). A 10-fold dilution series was used for each gene. Real-time PCR efficiency (*E*) = (10 − 1/slope) for 10-fold dilution series and % real-time PCR efficiency = (*E* − 1) × 100, if the standard deviation for the efficiencies determined using 10-fold dilution. The geometric mean of the efficiencies for the indicated genes were used for housekeeping gene efficiency. The fold change of expression of a gene was calculated using double delta CT calculations using the housekeeping gene GAPDH for normalization.

### Statistical analysis

Analyses of independent data were performed by Student’s unpaired two-tailed *t*-test and one-way ANOVA followed by Tukey’s test using GraphPad Prism 9.4.1. *p* < 0.05 was considered statistically significant. Independent variables in the study included HEV inoculum, immunosuppressive drugs, and ribavirin treatment. Dependent variables in the study included the outcome seen in HEV shedding, testosterone level, sperm characteristics, accessory gland tropism, and immune response.

## Results

### HEV is associated with the head region of sperm cells

**Figure 1.**
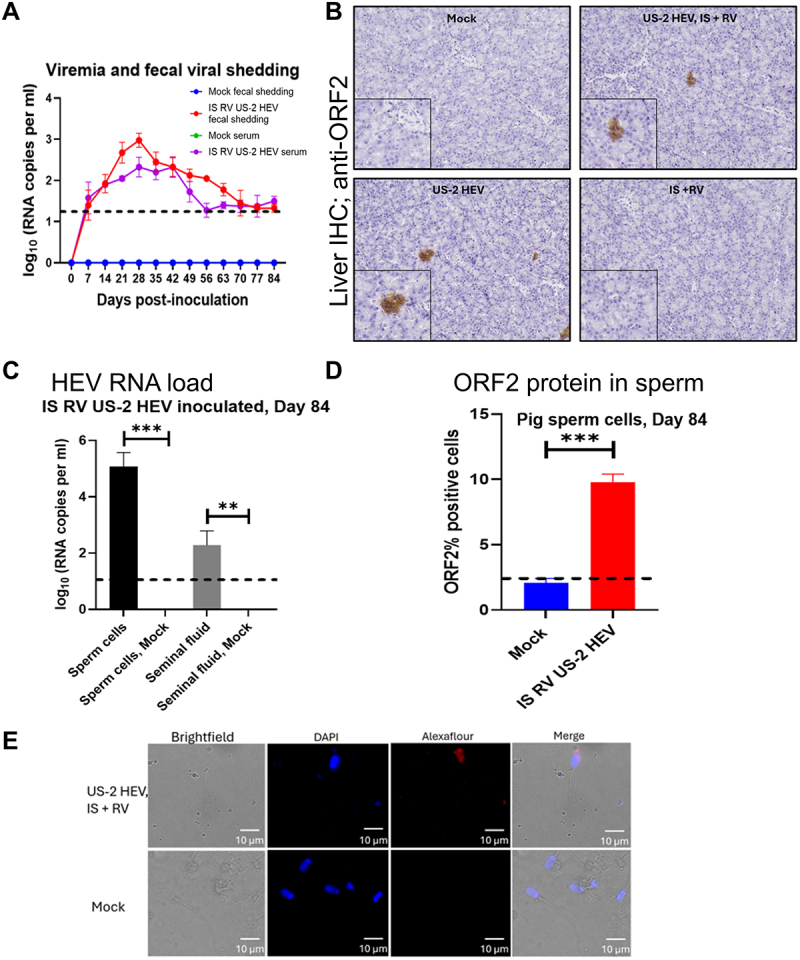
US-2 HEV-infected, immunosuppressed (IS), and ribavirin (RV)-treated sperm cells associate with hepatitis E virus.

In this study, we utilized the immunosuppressed pig model for chronic HEV infection [15]. Immunosuppressed pigs chronically infected with the *Paslahepevirus balayani* genotype 3 US-2 strain of HEV demonstrated viremia, HEV antigen in the liver, fecal virus shedding with low HEV titers until the study was terminated on d 84 (Figure 1(A,B)). Sperm cells separated from the seminal fluid of the US-2 HEV-infected immunosuppressed pigs demonstrated higher viral titers than that present in seminal fluid (Figure 1(C)). Using flow cytometry analysis, we report that at least 7% of the sperm cells from the chronically infected, immunosuppressed, and ribavirin-treated pigs contained HEV antigen (Figures 1(D) and 3(C)). Sperm cells collected from the immunosuppressed and ribavirin-treated pigs demonstrated the presence of HEV ORF2 antigen associated with the sperm head (acrosomal region), while the uninfected mock group demonstrated no HEV staining (Figure 1(E)).

(A) Viremia and fecal viral shedding from IS, RV-treated pigs inoculated with US-2 HEV. Mock-infected groups remained negative throughout the study. (B) IHC-stained liver of pig, showing HEV ORF2 protein (brown stain) in the US-2 HEV inoculated and US-2 HEV, IS + RV pigs. Mock and IS + RV pigs exhibit no IHC-positive cells. (C) HEV RNA loads in sperm cell suspension and spermatic fluid from IS RV-treated pigs inoculated with US-2 HEV. (D) Flow cytometry analysis showing the percentage of sperm cells containing hepatitis E virus (US-2 strain). (E) Immunohistochemical detection of hepatitis E (red) in the acrosomal region of sperm head obtained from the IS RV US-2 HEV-infected pigs at d 84 postinfection; HEV open-reading frame (ORF2) is red; DAPI stain is blue (nucleus). The dotted line represents the cutoff value demonstrating the background. **indicates *p* < 0.01, ***indicates *p* < 0.001, ****indicates *p* < 0.0001. IS – immunosuppressed, RV – ribavirin.

### Sperm cells collected from immunosuppressed US-2 HEV infected pigs are infectious to Huh7 S10-3 cells

**Figure 2.**
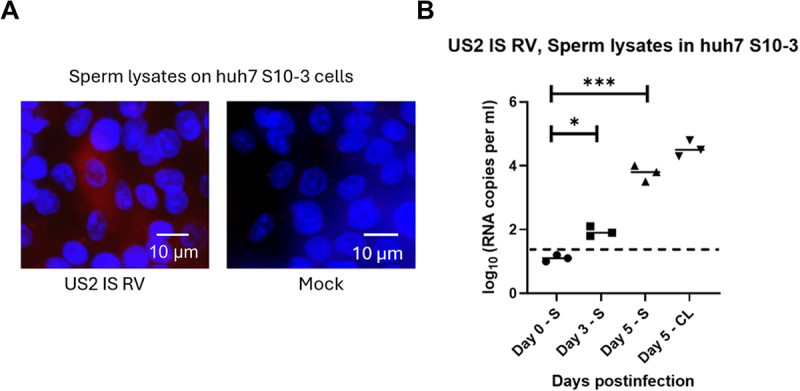
Sperm cells collected from the US-2 HEV-infected, immunosuppressed (IS) and ribavirin (RV)-treated pigs are infectious to Huh7 s10-3 cells.

Higher HEV RNA copies were seen in sperm cell lysates in comparison to the seminal fluid (Figure 1(B)). To evaluate the infectious ability of the HEV associated with the sperm cells, we lysed sperm (~6.2 × 10^5^ viral RNA copies) cells and inoculated human liver cells (Huh7 S10-3) *in vitro*. HEV ORF2 in Huh7 S10-3 cells was detected 5 d postinoculation with sperm cell lysates demonstrating the HEV associated with the sperm cells was viable virus (Figure 2(A)). Inoculated HEV was further shown to be replication-competent by an increase in viral RNA in supernatant harvested from the inoculated Huh7 S10-3 cells from d 0, 3, and 5 (Figure 2(B)).

(A) Immunodetection of HEV in Huh7 S10-3 cells inoculated with sperm cell lysate showing infectious virus in sperm. (B) Replication kinetics of sperm-derived HEV using Huh7 S10-3 cells. US-2 HEV RNA loads in culture supernatant (S) and cell lysates (CL) of Huh7 S10-3 cell cultures after inoculation with the lysed sperm cells collected from the IS RV US-2 HEV-inoculated pigs. Independent biological experiments, mean ± SD of three replicates, are presented. The dotted line represents the cutoff value demonstrating the background from initial attachment of the virus to the cell surfaces. **indicates *p* < 0.01, ***indicates *p* < 0.001.

### Sperm motility and morphology were altered by HEV infection in the immunosuppressed pigs

**Figure 3.**
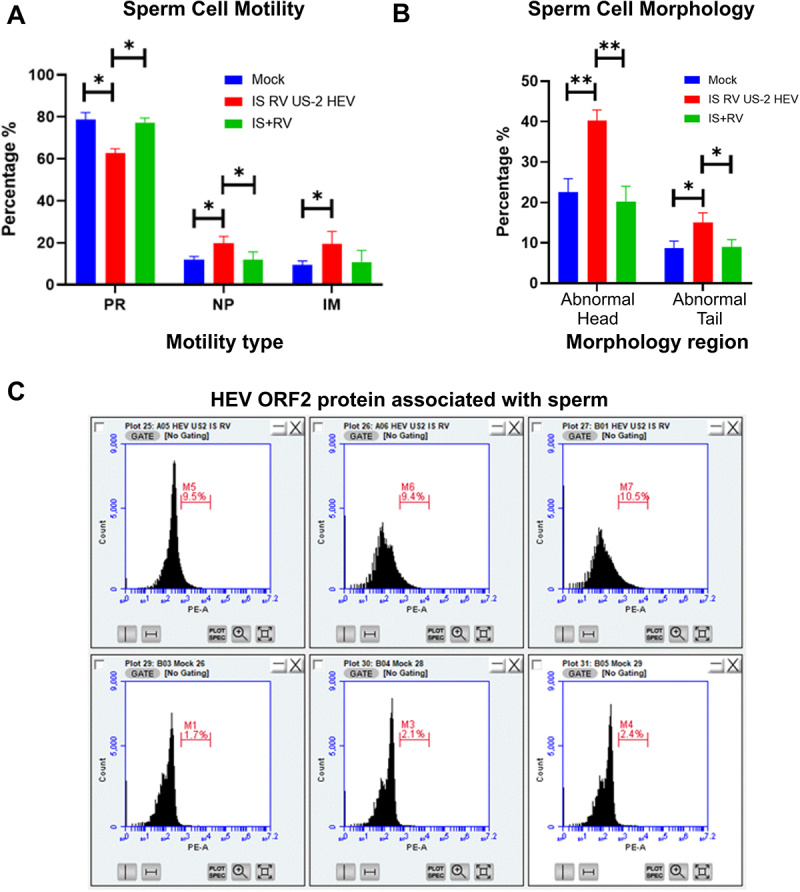
Hepatitis E virus alters mature sperm cell motility and morphology in immunosuppressed (IS), ribavirin (RV)-treated pigs.

Semen analysis was immediately performed after collection of semen from the epididymis. To characterize the motility and morphological difference between groups, 200 sperm cells were analyzed. The progressive sperm motility in the immunosuppressed, ribavirin-treated US-2 HEV-infected pigs (PR% = 62% ± 2%) was found to be significantly lower than the mock-infected pig sperm (78% ± 3%) and immunosuppressed, ribavirin-treated (77% ± 2%) pigs (Figure 3(A)). The nonprogressive sperm motility in immunosuppressed, ribavirin-treated US-2 HEV-infected pigs (NP% = 19% ± 5%) was found to be significantly higher than the mock-infected (12% ± 2%) and immunosuppressed, ribavirin-treated (10% ± 4%) pigs (Figure 3(A)). Immobility of sperm was higher in sperm from the immunosuppressed, ribavirin-treated US-2 HEV-infected pigs (19% ± 7%), when compared to mock-infected (10% ± 3%) and immunosuppressed, ribavirin-treated (13% ± 4%) pigs (Figure 3(A)). Morphologically, we found a higher percentage of abnormal sperm heads (40% ± 4%) in immunosuppressed, ribavirin-treated US-2 HEV-infected pigs when compared to mock infected (22% ± 3%) and immunosuppressed, ribavirin-treated (20% ± 4%) pigs (Figure 3(B)). We also found higher percentage of abnormal tails (15% ± 3%) in immunosuppressed, ribavirin-treated US-2 HEV-infected pigs when compared to mock infected (8% ± 2%) and immunosuppressed, ribavirin-treated (8% ± 2%) pigs (Figure 3(B)).

(A) Light microscopic observation of 200 live mature sperm cells harvested from mock, IS + RV, or IS, RV, and infected pig epididymis. Sperm cells demonstrated decreased progressive motility when infected by HEV US-2 in IS, RV-treated group. PR – progressive motility of sperm (moving active, either linearly or in a circle, regardless of speed); NP – nonprogressive motility (all other patterns of motility with absent progression). IM – immobility. (B) Light microscopic observation of live sperm cells harvested from mock, IS + RV, or IS, RV, and US-2 HEV infected pig epididymis. Sperm from US-2 HEV infected IS, RV-treated pigs showed a significant increase in mature sperm cells with head abnormalities. No significant changes were observed in the tail of the sperm cells. *indicates *p* < 0.05, **indicates *p* < 0.01. (C) A histogram plot was used to show the flow cytometry results. Sperm cells from mock noninfected pigs and from US-2 HEV infected, immunosuppressed (IS), and ribavirin (RV)-treated pigs.

### HEV infection leads to testicular damage through apoptosis and induces a hormonal disorder

**Figure 4.**
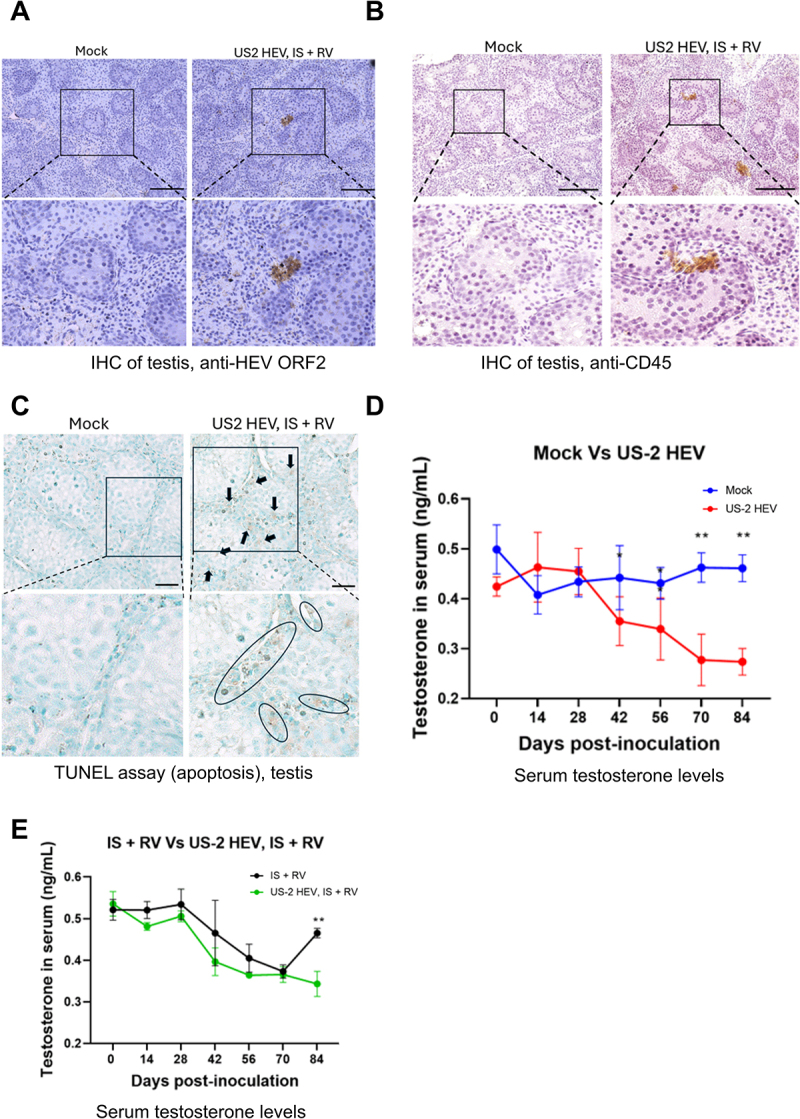
Hepatitis E virus replication, CD45+ leukocyte infiltration and apoptosis in blood–testis barrier (BTB).

The blood–testis barrier (BTB) protects the testis against invading pathogens and restricts immune cell entry to prevent immune-mediated sperm cell damage [25]. Apoptosis of the Sertoli cells forming the BTB leads to inflammatory changes and eventually causes a disturbance in the spermatogonia [26]. Immunohistochemistry of the testicles was performed to explore the mechanisms underlying the reductions in the sperm quality caused by HEV infection. HEV ORF2 antigen was observed at the BTB of pigs harvested at 84 d postinoculation (Figure 4(A)). The breakage of BTB caused by HEV infection may have accounted for the increased infiltration of the testis of HEV-infected pigs by CD45+ leukocytes seen at 84 dpi (Figure 4(B)). Terminal deoxynucleotidyl transferase biotin-dUTP nick end labeling (TUNEL) assays revealed the apoptosis of cells bordering the BTB (Figure 4(C)). Serum testosterone concentrations were significantly decreased in US-2 HEV-infected pigs tested on d 70 and 84 postinoculation compared to mock pigs (Figure 4(D)). Interestingly, pigs under immunosuppressive and ribavirin drug treatment also demonstrated a decrease in the serum testosterone concentration similar to the US-2 HEV-infected, immunosuppressed, and ribavirin-treated pigs (Figure 4(E)). However, on d 84, a significantly lower level of testosterone was observed only in US-2 HEV-infected, immunosuppressed, and ribavirin-treated pigs (Figure 4(E)).

(A) Immunohistochemical (IHC) staining of testis, demonstrating the HEV open reading frame (ORF) 2 capsid protein and infiltration of CD45+ leukocytes (B) at the BTB in US-2 HEV-infected, immunosuppressed (IS), and ribavirin (RV)-treated pigs. (C) TUNEL assay demonstrating apoptosis at the BTB of US-2 HEV-infected, immunosuppressed (IS), and ribavirin (RV)-treated pigs. (D) Serum testosterone levels measurement between mock and US-2 HEV-infected pigs demonstrates significant decrease in the virus-infected group on d 70 and 84. (E) Serum testosterone level between immunosuppressed (IS) + ribavirin (RV) and US-2 HEV-infected, IS, and RV-treated pigs were similar except on d 84.

### HEV replication in the male accessory glands

To evaluate the contribution of the male accessory glands in HEV reproductive pathogenesis, persistence, and contribution to semen, we utilized immunocompetent and immunocompromised pigs. HEV antigen was demonstrated in the prostate gland, Cowper’s gland, seminal vesicle, and epididymis on d 84 postinoculation (Figure 5(A)). HEV viral RNA was higher in the prostate gland, followed by seminal vesicle, and Cowper’s gland in immunocompetent pigs compared to chronically infected immunosuppressed pigs (Figure 5(B)). Moreover, we saw less HEV antigen in the immunocompromised pigs when compared to immunocompetent pigs. Mock-infected pigs and mock-infected, immunosuppressed, and ribavirin-treated pigs did not demonstrate HEV RNA and/or Ag in the male accessory glands. Infiltration of CD45+ leukocytes was evident in the prostate gland, seminal vesicles, and epididymis of the US-2 HEV-infected pigs on d 84 postinoculation. In contrast, US-2 HEV-infected, immunosuppressed, and ribavirin-treated pigs demonstrate less infiltration of the CD45+ leukocytes on d 84 postinoculation (Figure 5(C)). A TUNEL assay revealed higher apoptosis in the prostate gland, seminal vesicles, and epididymis of US-2 HEV-infected pigs than in the US-2 HEV-infected, immunosuppressed, and ribavirin-treated pigs on d 84 postinoculation (Figure 5(D)).

**Figure 5.**
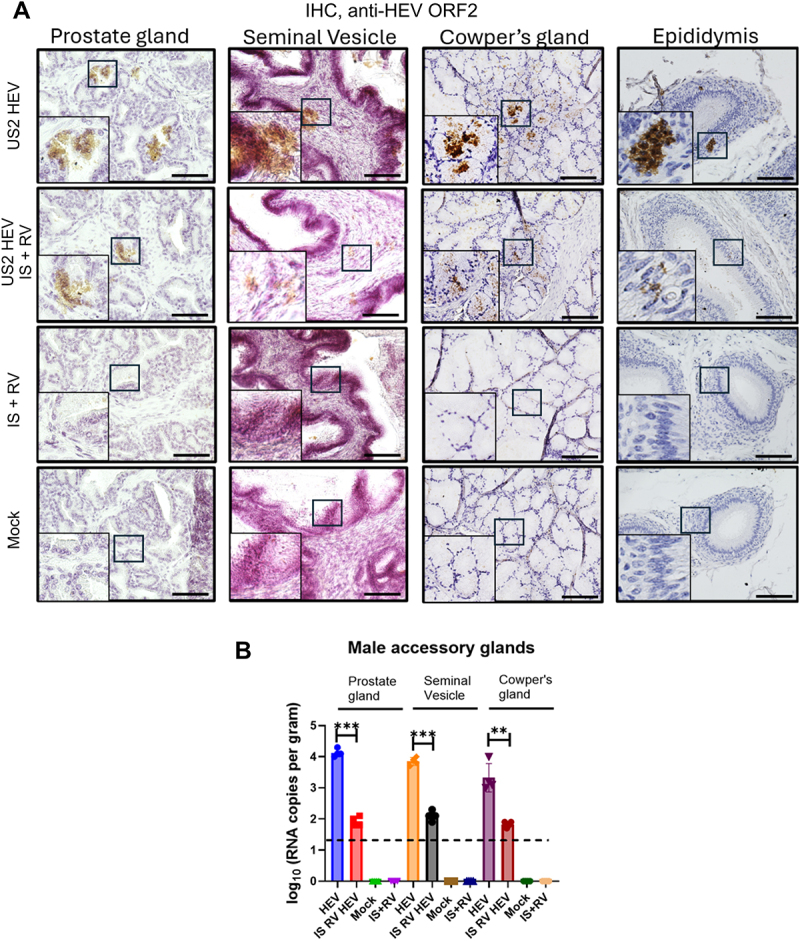
HEV infection and apoptosis in the male accessory glands.

(A) Immunohistochemical (IHC) staining of prostate, seminal vesicles, Cowper’s gland, and epididymis demonstrating the HEV open reading frame (ORF) 2 capsid protein. (B) Viral RNA loads in the prostate gland, seminal vesicles, and Cowper’s glands of US-2 HEV-infected (HEV); IS, RV, US-2 HEV-infected (IS RV HEV); mock, and IS + RV. The dotted line represents the cutoff value demonstrating the background. **indicates *p* < 0.01, ***indicates *p* < 0.001. (C) Infiltration of CD45+ leukocytes in prostate, seminal vesicle, Cowper’s gland, and epididymis between all four groups. (D) TUNEL assay demonstrating apoptosis in the prostate, seminal vesicle, Cowper’s gland, and epididymis between all four groups. HEV – hepatitis E virus, IS – immunosuppressed, RV – ribavirin.

**Figure 5.**
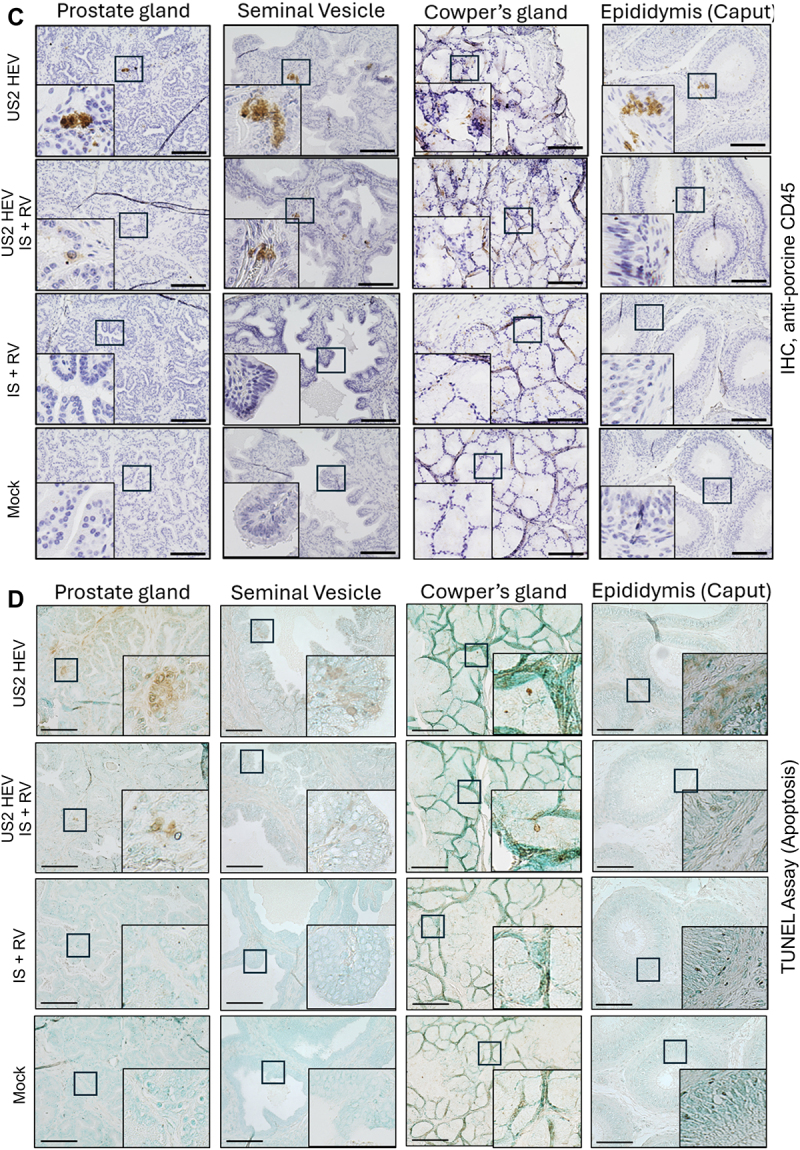
(Continued).

## Discussion

The presence of RNA virus in the head [27] and tail region [22] of sperm cells has been demonstrated for ZIKA virus, revealing its ability and mode of sexual transmission in humans [28]. The presence of infectious HEV in the sperm head is a very interesting finding, highlighting a potential for sexual transmission of HEV [13]. Our recent findings have shifted the paradigm of hepevirus–host interaction and highlight the need to understand possible sexual transmission of HEV.

Here, we demonstrate high viral RNA copies in sperm cells from immunosuppressed, ribavirin-treated pigs. Our study necessitates an understanding of viral persistence in sperm cells after symptomatic and asymptomatic HEV infections. Infectious HEV seen in the sperm cells collected on d 84 suggests the need to understand the length of infectious virus persistence in sperm cells. Recent reports from chronically infected patients also demonstrated infectious HEV particles in the ejaculate [10] as demonstrated in our immunosuppressed, ribavirin-treated US-2 HEV-infected pigs suggesting immunosuppressed pigs can be used as an efficient model to study the HEV effect on sperm health. Pigs being the natural host for HEV gt3 in addition to the presence of similar accessory glands (prostate, seminal vesicle, and Cowper’s gland) provides an advantage to explore the HEV shedding in ejaculates. Other viruses such as Zika and Ebola have been studied for sexual transmission between humans and have demonstrated a long-lasting excretion of viral RNA in the semen [29,30]. Zika virus has also been shown to cause a decrease in sperm motility and morphology in humans [31]. A decrease in sperm motility has been reported in Zika-virus-infected animals [32,33] and associated with infertility in humans [34]. Similarly, our study demonstrated a decrease in the progressive motility of sperm cells in immunosuppressed chronically infected pigs. Previously, we reported 19% of the sperm cells associated with HEV ORF2 antigen in US-2 HEV-infected pigs with high viral RNA titers [13]. Interestingly, in the chronically infected, immunosuppressed, and ribavirin-treated pigs, only 7% of the sperm cells (absolute positive by deducting mock-positive staining background) were positive for HEV ORF2, demonstrating a significant difference and showed comparatively lower viral titers. We speculate ribavirin treatment to be one of the major factors directly associated with our observed decrease of the virus in the sperm cells. Ribavirin is known to cross the BTB in humans [35] and rats [36] and thus we hypothesize that it may be playing a crucial role affecting the viral replication in the testis of pigs. Interestingly, azathioprine, used in our study, has been shown recently to cause impairment in the BTB, spermatogenesis, and is also known to disrupt apoptosis-related gene expression [37]. This could partially explain some trends of decreased apoptosis gene expression that we see with the BAX and PARP1 genes in immunosuppressed tissues (Supplementary data 1, Figure S1 and S2) though it appears only analyzing a small piece of the entire infected tissue introduced substantial variability making significant conclusions difficult. We also saw an increased expression of CASP3 genes in the US-2 HEV-infected, immunosuppressed, and ribavirin-treated groups (Supplementary data 1, Figure S3), which could explain the combined pathological outcome; however, limitation in the number of animals restricts us in drawing a major conclusion. Thus, future studies need to be directed toward understanding the transport of ribavirin across the BTB in pigs, the effect of immunosuppressive drugs such as azathioprine and role of HEV persistence and its effect in HEV quasispecies formation and overall impact in spermatogenesis.

Multiple viruses disrupt the BTB by various mechanisms. For instance, zika virus infection affects the permeability of the BTB through matrix metalloproteinase 9 (MMP9)-mediated degradation of tight-junction proteins and type IV collagens that are involved in the maintenance of BTB [38]. HIV-1 disrupts the BTB permeability by utilizing its Tat protein transduction domain [39]. Mumps and SARS-CoV-2 disrupt BTB via decreasing the expression of junctional proteins, such as occludin, claudin-11 [40,41]. In our study, we demonstrate the damage in the BTB integrity during HEV infection. Apoptosis and infiltration of CD45+ leukocytes were evident at the BTB of chronically infected pigs, while no significant apoptosis was present in chronically immunosuppressed, uninfected group (Supplementary data 1, Fig. S4). A recently published study suggested HEV possessed the ability to infect Sertoli cells and alter the cytokine milieu [42]. Thus, we hypothesize that HEV disruption of the BTB leading to apoptosis could be either due to downexpression or degradation of the junction proteins. It would be interesting to study the effects of the HEV viral protein in the expression of the BTB junctional proteins. Future research understanding the mechanism of BTB disruption by HEV is warranted.

Leydig cells are known as the primary source of testosterone [43] and are located just adjacent to the BTB [44]. Decreases in serum testosterone are directly related to a decrease in the numbers of Leydig cells [45]. In our study, US-2 HEV-infected pigs showed a decrease in serum testosterone levels on d 70 and 84 indicating a decrease in Leydig cells. We also saw a decrease in testosterone levels at d 42, 56, and 70 in the immunosuppressed ribavirin-treated group and immunosuppressed, ribavirin-treated, and HEV-infected group, which could be an effect of azathioprine in BTB and spermatogenesis [37]. Testosterone concentration has been demonstrated to affect spermatogenesis. In the absence of testosterone signaling: (1) mature spermatozoa are retained within the seminiferous tubules and phagocytized by Sertoli cells [46,47], (2) BTB tight-junction protein assembly rate is decreased [48], (3) the amount of connexin between the Sertoli cells and male germ cells is reduced, leading to premature separation of round spermatids from Sertoli cells [49]. In our HEV-infected pig model, abnormal sperm morphology was documented in addition to apoptosis at the BTB. We speculate that HEV infection in the testicular tissues and significant decrease in the serum testosterone concentration causes disturbance in testicular homeostasis leading to decreased progressive motility as reported in our study.

In general, infection of the reproductive system activates the innate immune system. This response can disturb the testis-specific restricted immunological response leading to leukocyte infiltration and resulting in oxidative stress responsible for the damage to the testicular tissues and compromising the BTB integrity [50,51]. Infiltration of CD45+ cells were observed in the chronically infected immunosuppressed pigs. CD45+ lymphocyte infiltration is directly associated with abnormal sperm morphology and early apoptosis of the sperm cells [52,53]. Infiltration of CD45 in HEV-infected mice testis has been attributed to the breakage of the BTB caused by HEV infection [16]. Severe inflammation as indicated by CD45+ cell infiltration and azathioprine could be playing an important role in disruption of immune privilege leading to reduced sperm motility, and increased sperm abnormalities among HEV-infected males. A long-term study demonstrating the viral clearance, status of BTB, immune cells, and sperm quality report will be needed to understand if the resolution is partial or full after HEV infection.

Sexual transmission of HEV in humans has not yet been documented. We speculate this may be due to male partners of females demonstrating HEV infection are not routinely screened for HEV in their semen samples. Considering, the presence of viral RNA in chronic patients [9], viral RNA in the semen of infertile patients [11], and experimental studies demonstrating the infectious HEV in the ejaculate [10] and sperm head [13], further research is needed in larger cohorts to determine the contribution of a sexual route of transmission in human patients. In addition to HEV gt4 infection in testis of rhesus macaques, mice [16] and gerbils [17], and gt3 in pigs (our study) HEV gt1 infection in testis has been reported utilizing the immunocompetent gerbil model [54] suggesting HEV tropism to the testis could be a shared character between different genotypes that needs further analysis. Screening of male patients in HEV-endemic areas is very much essential to determine the effect of HEV on sperm quality and reproductive health disorders.

Association of HEV with the female reproductive system has been studied for decades to understand the pathogenesis behind pregnancy mortality, but the mechanism by which HEV alters male fertility still awaits in-depth explanation. Recent literature suggests a new dynamic of HEV to the male genital system. HEV replication in human Sertoli cells [42], association with the sperm head in the pig model [13], and transmission to the liver through the intravaginal route in the rabbit model [55] necessitates study of the nature and mechanisms of HEV host interactions in the male genital tract. Semen is a mixture of sperm cells and secreting fluids; thus, the presence of virus in the semen can be due to viral replication in the male accessory glands [56]. Interestingly, the most studied tissues (testis and epididymis) of the male reproductive tract only contribute 10% to the semen volume [57]. The major portion of the semen is contributed by the seminal vesicles (65–75%) and the prostate gland (25–30%) [57]. Sperm parameters are directly influenced by the infection in the male accessory glands. Measuring the specific secretary products of these glands in the seminal fluid will allow us to understand the effect of one gland in comparison to others [58]. However, they have not been studied well for their role in viral replication. The most studied RNA virus, HIV, has been demonstrated to infect seminal vesicles both *in vitro* and *in vivo* [59]. Simian immunodeficiency virus (SIV) and Zika virus findings in male accessory glands demonstrate the importance of the prostate and seminal vesicles as a potential reservoir for persistent infection [60–62]. In our study, HEV has been demonstrated in the male accessory glands, mainly prostate, seminal vesicle, and epididymis of pigs harvested on d 84 postinoculation, suggesting HEV replication in these accessory glands. It would be interesting to study whether HEV can still be present in seminal fluids after vasectomy. These studies would further define the role of the accessory glands in the contribution of HEV in the semen. Additionally, the presence of leukocytes in the male accessory glands of pigs is a sign of infection and/or localized inflammatory response. It would be interesting to investigate the leukocytospermia and HEV infection-associated effects in the sperm quality and overall reproductive health. A positive association between leukocytospermia, ROS, and sperm DNA fragmentation has been described [63]. We speculate that the presence of leukocytes in the male accessory glands and testis could be a contributing factor to low progressive motility seen in HEV-infected pigs.

The virus shedding in the semen also depends on viral attachment to the specific receptors present in the spermatozoa. In the case of HIV, alternative receptors, such as heparan sulfate and Ga1AAg (glycolipid-related glyceramide), have been demonstrated to play an important role in the capture of HIV [64]. Interestingly, one of the receptors defined for HEV is heparan sulfate [65]. Studies understanding if heparan sulfate of sperm cells have a role in the capture of HEV will be an interesting topic. As HIV has been studied for decades, semen has been considered as the main vector for the HIV-1 dissemination. Three major sources of infectious HIV have been reported: free virions, infected leukocytes, and spermatozoa-associated virions [66]. Future studies characterizing the presence of HEV in the semen following the similar differentiation is essential to understand the duration of infectious HEV present in the semen.

Some limitations of these studies include a lack of testis tissues from the immunocompetent pigs which was lost during sample processing. Further understanding the in-depth immunological response in specific tissues of the male reproductive system would give us a clearer picture of the host immunological response during chronicity, and we anticipate undertaking this research in future studies. Some pigs would regurgitate the immunosuppressive drugs during oral feeding, thus possibly contributing to individual differences in the virus titer possibly impacting the overall virus presence in the sperm cells and MRT. We do not know the amount of ribavirin crossing the BTB in these pigs, necessitating further pharmacokinetic studies to understand and compare RBV entry into the testes between humans and pigs to further validate the model.

## Conclusion

In conclusion, immunosuppressed ribavirin-treated and infected pigs demonstrated the infectious HEV in the sperm head with low titers when compared to nonimmunosuppressed HEV-infected pigs. Our in-depth examination of the pig male accessory glands revealed the presence of HEV in the testis, in addition to the infiltration of CD45 cells and revealing some typical characteristics of apoptosis. Nonimmunosuppressed HEV-infected pigs have HEV antigen distributed throughout the accessory glands but immunosuppressed ribavirin-treated HEV-infected pigs demonstrated HEV antigen mainly in the prostate gland. Collectively, this experimental infection in immunosuppressed, ribavirin-treated recapitulates the scenario of HEV-positive semen seen in chronic human patients [10] and thus paves a new path to understand the role of accessory glands in the maintenance of HEV during chronic HEV infection.

## Supplementary Material

Supp_S4_TUNEL.jpg

Figure Legend All 07012026.docx

ARRIVE Author Checklist_Full_spk_10092025.pdf

Supp_S2_PARP1.jpg

Supplementary_Data_1_02142026.docx

Supp_S1_BAX.jpg

Supp_S3_CASP3.jpg

## Data Availability

Raw data were generated at The Ohio State University. The data that support the findings of this study are openly available on Figshare at https://doi.org/10.6084/m9.figshare.28528151 [68].
