# Supplementary figures and images for: A porcine model of chronic hepatitis E virus (HEV) infection identifies male reproductive glands as sites of viral persistence
Source: Virulence. 2026 Jul 22;17(1):2707701. doi: 10.1080/21505594.2026.2707701 (PMC13432849; doi:10.1080/21505594.2026.2707701)

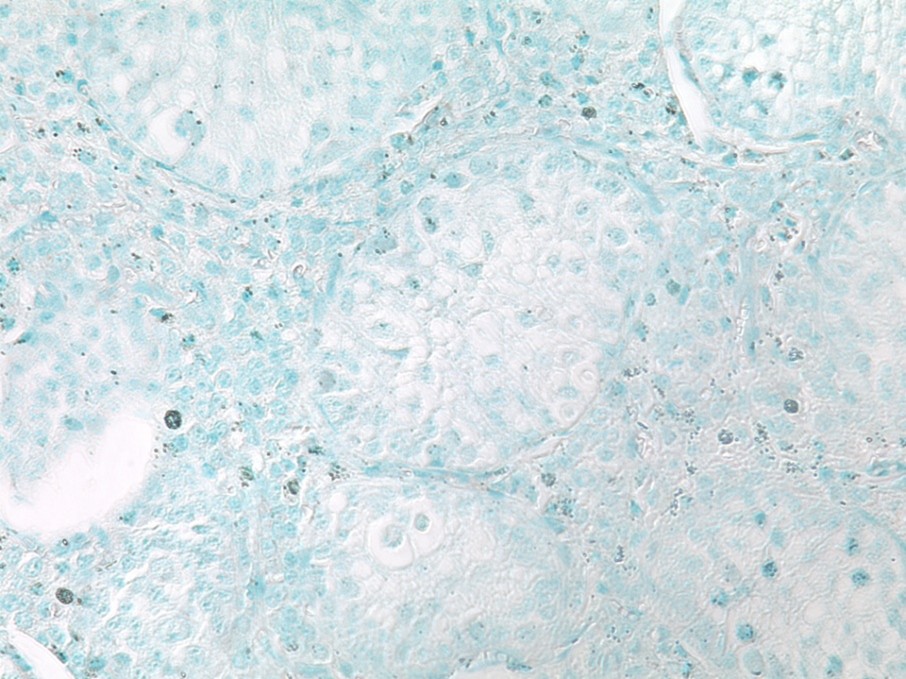

Supplement: Supp_S4_TUNEL.jpg [file KVIR_A_2707701_SM0840.jpg]

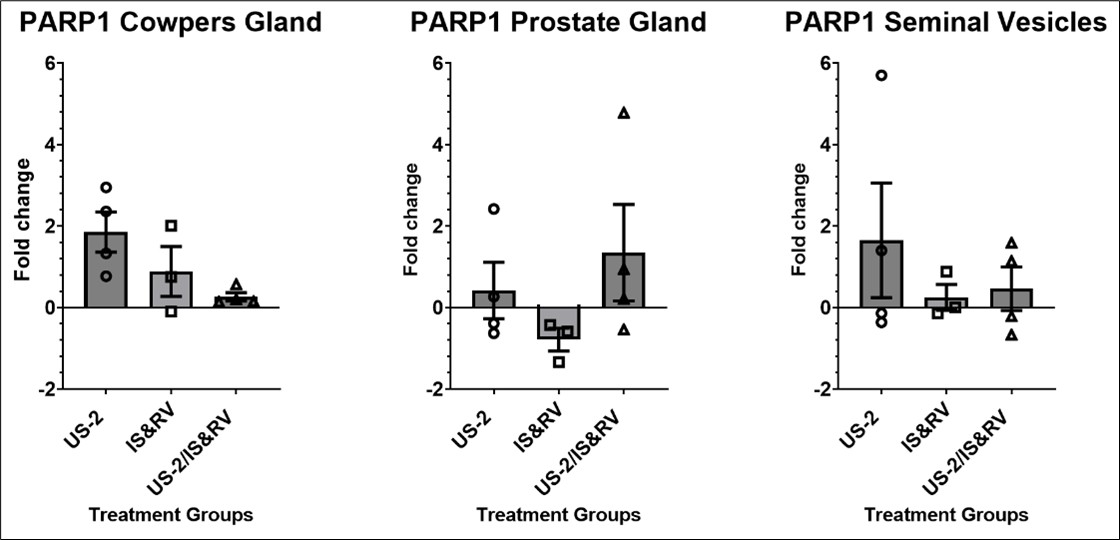

Supplement: Supp_S2_PARP1.jpg [file KVIR_A_2707701_SM0837.jpg]

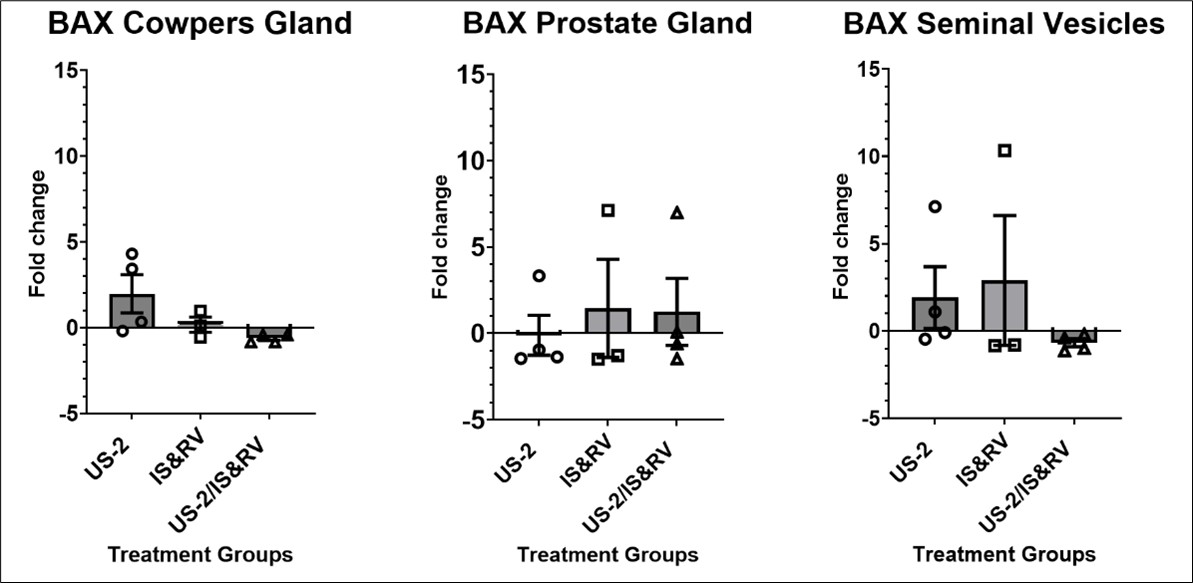

Supplement: Supp_S1_BAX.jpg [file KVIR_A_2707701_SM0835.jpg]

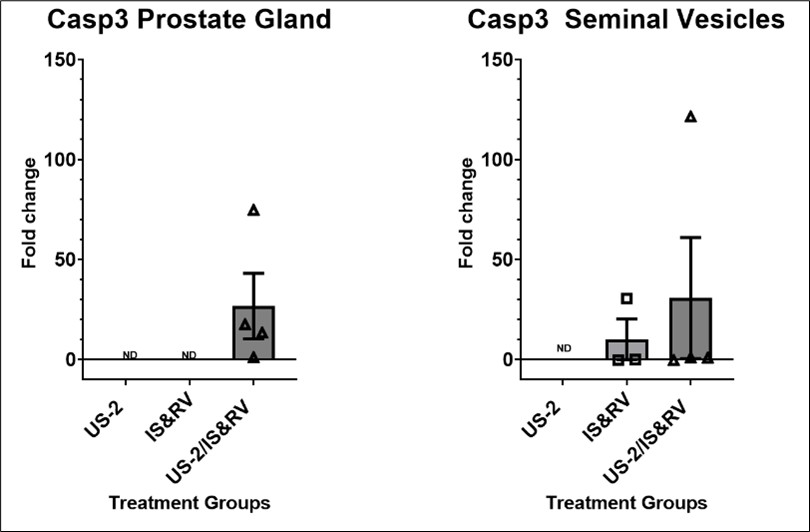

Supplement: Supp_S3_CASP3.jpg [file KVIR_A_2707701_SM0834.jpg]
