# Supplementary material for: A porcine model of chronic hepatitis E virus (HEV) infection identifies male reproductive glands as sites of viral persistence
Source: Virulence. 2026 Jul 22;17(1):2707701. doi: 10.1080/21505594.2026.2707701 (PMC13432849; doi:10.1080/21505594.2026.2707701)
Supplement: Figure Legend All 07012026.docx [file KVIR_A_2707701_SM0839.docx]

**Figure 1.** US-2 HEV infected, immunosuppressed (IS) and ribavirin (RV) treated sperm cells associate with hepatitis E virus.

**Fig 1A.** Viremia and fecal viral shedding from IS, RV treated pigs inoculated with US-2 HEV. Mock-infected groups remained negative throughout the study.

**Fig 1B.** IHC – stained liver of pig, showing HEV ORF2 protein (brown stain) in the US-2 HEV inoculated and US-2 HEV, IS + RV pigs. Mock and IS + RV pigs exhibit no IHC-positive cells.

**Fig1C.** HEV RNA loads in sperm cells suspension and spermatic fluid from IS RV treated pigs inoculated with US-2 HEV.

**Fig 1D.** Flow cytometry analysis showing the percentage of sperm cells containing hepatitis E virus (US-2 strain).

**Fig 1E.** Immunohistochemical detection of hepatitis E (red) in the acrosomal region of sperm head obtained from the IS RV US-2 HEV infected pigs at day 84 post infection; HEV open reading frame (ORF2) is red; DAPI stain is blue (nucleus).

The dotted line represents the cut-off value demonstrating the background. ** indicates p < 0.01, *** indicates p < 0.001, **** indicates p < 0.0001. IS – immunosuppressed, RV – ribavirin.

**Figure 2.** Sperm cells collected from the US-2 HEV infected, immunosuppressed (IS) and ribavirin (RV) treated pigs are infectious to Huh7 s10-3 cells.

**Fig 2A.** Immunodetection of HEV in Huh7 S10-3 cells inoculated with sperm cell lysate showing infectious virus in sperm.

**Fig 2B.** Replication kinetics of sperm derived HEV using Huh7 S10-3 cells. US-2 HEV RNA loads in culture supernatant (S) and cell lysates (CL) of Huh7 S10-3 cell cultures after inoculation with the lysed sperm cells collected from the IS RV US-2 HEV inoculated pigs. Independent biological experiments, mean ± SD of three replicates, are presented. The dotted line represents the cut-off value demonstrating the background from initial attachment of the virus to the cell surfaces. ** indicates p < 0.01, *** indicates p < 0.001.

**Figure 3.** Hepatitis E virus alters mature sperm cell motility and morphology in immunosuppressed (IS), ribavirin (RV) treated pigs.

**Fig 3A.** Light microscopic observation of 200 live mature sperm cells harvested from mock, IS + RV, or IS, RV and infected pig epididymis. Sperm cells demonstrated decreased progressive motility when infected by HEV US-2 in IS, RV treated group. PR – progressive motility of sperm (moving active, either linearly or in a circle, regardless of speed); NP – non-progressive motility (all other patterns of motility with absent progression). IM – immobility.

**Fig 3B.** Light microscopic observation of live sperm cells harvested from mock, IS + RV, or IS, RV and US-2 HEV infected pig epididymis. Sperm from US-2 HEV infected IS, RV treated pigs showed a significant increase in mature sperm cells with head abnormalities. No significant changes were observed in the tail of the sperm cells. * indicates p < 0.05, ** indicates p < 0.01.

**Fig 3C.** A histogram plot was used to show the flow cytometry results. Sperm cells from mock non-infected pigs and from US-2 HEV infected, immunosuppressed (IS) and ribavirin (RV) treated pigs.

**Figure 4.** Hepatitis E virus replication, CD45+ leukocyte infiltration and apoptosis in blood testis barrier (BTB).

**Fig 4A.** Immunohistochemical (IHC) staining of testis demonstrating the HEV open reading frame (ORF) 2 capsid protein and infiltration of CD45+ leukocytes

**Fig 4B.** Immunohistochemical (IHC) staining of testis demonstrating the HEV open reading frame (ORF) 2 capsid protein and infiltration of CD45+ leukocytes at the BTB in US-2 HEV infected, immunosuppressed (IS) and ribavirin (RV) treated pigs.

**Fig 4C.** TUNEL assay demonstrating apoptosis at the BTB of US-2 HEV infected, immunosuppressed (IS) and ribavirin (RV) treated pigs.

**Fig 4D.** Serum testosterone levels measurement between mock and US-2 HEV infected pigs demonstrates significant decrease in the virus infected group on day 70 and 84.

**Fig 4E.** Serum testosterone level between immunosuppressed (IS) + ribavirin (RV) and US-2 HEV infected, IS and RV treated pigs were similar except on day 84.

**Figure 5.** HEV infection and apoptosis in the male accessory glands.

**Fig 5A.** Immunohistochemical (IHC) staining of prostate, seminal vesicles, Cowper’s gland and epididymis demonstrating the HEV open reading frame (ORF) 2 capsid protein.

**Fig 5B.** Viral RNA loads in the prostate gland, seminal vesicles and Cowper’s glands of US-2 HEV infected (HEV); IS, RV, US-2 HEV infected (IS RV HEV); mock, and IS + RV. The dotted line represents the cut-off value demonstrating the background. ** indicates p < 0.01, *** indicates p < 0.001.

**Fig 5C.** Infiltration of CD45+ leukocytes in prostate, seminal vesicle, Cowper’s gland and epididymis between all four groups.

**Fig 5D.** TUNEL assay demonstrating apoptosis in the prostate, seminal vesicle, Cowper’s gland and epididymis between all four groups. HEV – Hepatitis E virus, IS – Immunosuppressed, RV - Ribavirin.

**Supp. S1** BAX gene quantification is performed in Cowper’s gland, Prostate gland, Seminal Vesicles.

**Supp. S2** PARP1 gene quantification is performed in Cowper’s gland, Prostate gland, Seminal Vesicles.

**Supp. S3** CASP3 gene quantification is performed in Prostate gland, and Seminal Vesicles.

**Supp. S4** TUNEL assay performed in immunosuppressed and untreated group testis.

**Table 1.** Experimental groups in the study.
