# Supplementary material for: A porcine model of chronic hepatitis E virus (HEV) infection identifies male reproductive glands as sites of viral persistence
Source: Virulence. 2026 Jul 22;17(1):2707701. doi: 10.1080/21505594.2026.2707701 (PMC13432849; doi:10.1080/21505594.2026.2707701)
Supplement: Supplementary_Data_1_02142026.docx [file KVIR_A_2707701_SM0836.docx]

**Supplementary data 1**

**Objective 1:** To quantify BAX gene in Cowper’s gland, Prostate gland and Seminal Vesicles.


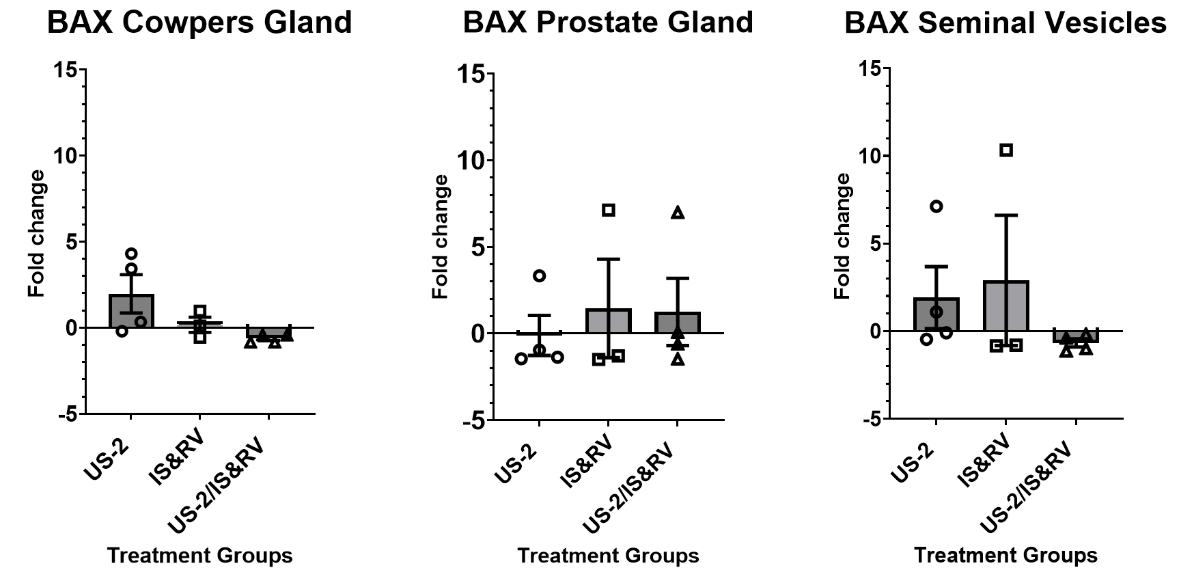


**Figure. S1.** BAX gene quantification is performed in Cowper’s gland, Prostate gland, Seminal Vesicles. The experiment was performed 3 times. Mock values have been subtracted from treatment groups.

**Results:** We could not see any major significant differences between the groups either with prostate, Cowper’s or seminal vesicles. A trend was seen in the Cowper’s gland with higher regulation of BAX gene in US-2 infected pigs but was insignificant.

**Objective 2:** To quantify PARP1 gene in Cowper’s gland, Prostate gland and Seminal Vesicles.


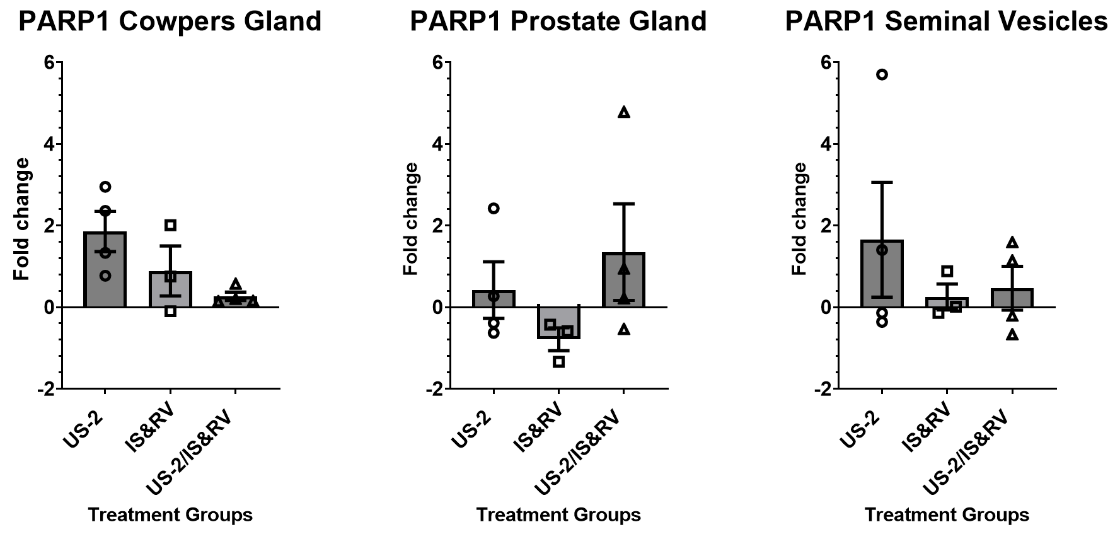


**Figure S2.** PARP1 gene quantification is performed in Cowper’s gland, Prostate gland, Seminal Vesicles. The experiment was performed 3 times. Mock values have been subtracted from treatment groups.

**Results:** We could not see any major significant differences between the groups either with prostate, Cowper’s or seminal vesicles. A trend was seen in the Cowper’s gland with higher regulation of BAX gene in US-2 infected pigs but was insignificant. We could also see a trend in seminal vesicles where PARP1 gene was highly expressed in US-2 infected pigs. However, we could see high expression of PARP1 gene in prostate gland of US2/IS&RV group.

**Objective 3:** To quantify CASP3 gene in Cowper’s gland, Prostate gland and Seminal Vesicles.


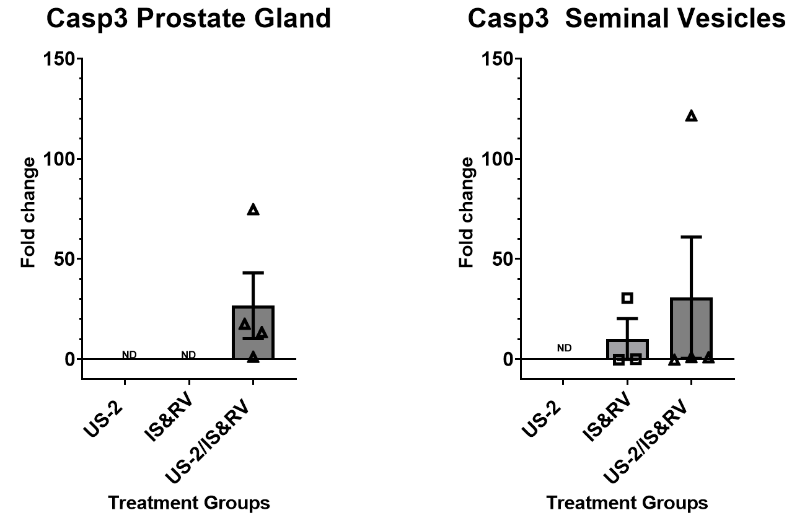


**Figure S3.** CASP3 gene quantification is performed in Prostate gland, and Seminal Vesicles. The experiment was performed 3 times. However, no quantification of CASP3 was detected in Cowper’s gland. Mock values have been subtracted from treatment groups.

**Results:** We could not see any major significant differences between the groups either with prostate, or seminal vesicles. We could see a trend in seminal vesicles but was not significant.

**Raw data from which the above graphs were made:**

| **BAX** |  |  | **PARP1** |  |  | **CASP3** |  |  |
| --- | --- | --- | --- | --- | --- | --- | --- | --- |
| **Cowpers Gland** |  |  | **Cowpers Gland** |  |  | **Cowpers Gland** |  |  |
| Animal | Expression |  | Animal | Expression |  | Animal | Expression |  |
| 1 | 3.43 | US-2 | 1 | 2.36 | US-2 | 1 | nd | US-2 |
| 2 | 4.31 |  | 2 | 2.95 |  | 2 | nd |  |
| 3 | 0.34 |  | 3 | 0.77 |  | 3 | nd |  |
| 4 | -0.20 |  | 4 | 1.33 |  | 4 | nd |  |
| 18 | -0.56 | IS&RV | 18 | -0.10 | IS&RV | 18 | nd | IS&RV |
| 19 | 0.11 |  | 19 | 0.75 |  | 19 | nd |  |
| 20 | 0.97 |  | 20 | 2.01 |  | 20 | nd |  |
| 33 | -0.43 | US-2/IS&RV | 33 | 0.57 | US-2/IS&RV | 33 | nd | US-2/IS&RV |
| 34 | -0.84 |  | 34 | 0.15 |  | 34 | nd |  |
| 35 | -0.84 |  | 35 | 0.14 |  | 35 | nd |  |
| 36 | -0.41 |  | 36 | 0.20 |  | 36 | nd |  |
| **Prostate** |  |  | **Prostate** |  |  | **Prostate** |  |  |
| Animal |  |  | Animal |  |  | Animal |  |  |
| 1 | -0.95 | US-2 | 1 | 0.27 | US-2 | 1 | nd | US-2 |
| 2 | -1.45 |  | 2 | -0.63 |  | 2 | nd |  |
| 3 | -1.36 |  | 3 | -0.39 |  | 3 | nd |  |
| 4 | 3.34 |  | 4 | 2.42 |  | 4 | nd |  |
| 18 | 7.13 | IS&RV | 18 | -1.34 | IS&RV | 18 | nd | IS&RV |
| 19 | -1.49 |  | 19 | -0.59 |  | 19 | nd |  |
| 20 | -1.29 |  | 20 | -0.43 |  | 20 | nd |  |
| 33 | 0.08 | US-2/IS&RV | 33 | 0.94 | US-2/IS&RV | 33 | 13.35 | US-2/IS&RV |
| 34 | -0.60 |  | 34 | 0.21 |  | 34 | 74.71 |  |
| 35 | -1.47 |  | 35 | -0.54 |  | 35 | 1.07 |  |
| 36 | 6.99 |  | 36 | 4.78 |  | 36 | 17.54 |  |
| **Seminal Vesicle** |  |  | **Seminal Vesicle** |  |  | **Seminal Vesicle** |  |  |
| Animal |  |  | Animal |  |  | Animal |  |  |
| 1 | 7.13 | US-2 | 1 | 5.70 | US-2 | 1 | nd | US-2 |
| 2 | -0.46 |  | 2 | -0.36 |  | 2 | nd |  |
| 3 | 1.10 |  | 3 | 1.40 |  | 3 | nd |  |
| 4 | -0.09 |  | 4 | -0.14 |  | 4 | nd |  |
| 18 | 10.33 | IS&RV | 18 | -0.14 | IS&RV | 18 | -0.08 | IS&RV |
| 19 | -0.80 |  | 19 | 0.01 |  | 19 | 30.47 |  |
| 20 | -0.84 |  | 20 | 0.88 |  | 20 | -0.24 |  |
| 33 | -0.99 | US-2/IS&RV | 33 | -0.21 | US-2/IS&RV | 33 | 0.89 | US-2/IS&RV |
| 34 | -0.18 |  | 34 | 1.59 |  | 34 | 121.48 |  |
| 35 | -0.39 |  | 35 | 1.14 |  | 35 | 0.80 |  |
| 36 | -1.13 |  | 36 | -0.67 |  | 36 | -0.36 |  |

Representative image of TUNEL assay in chronically immunosuppressed and untreated group.


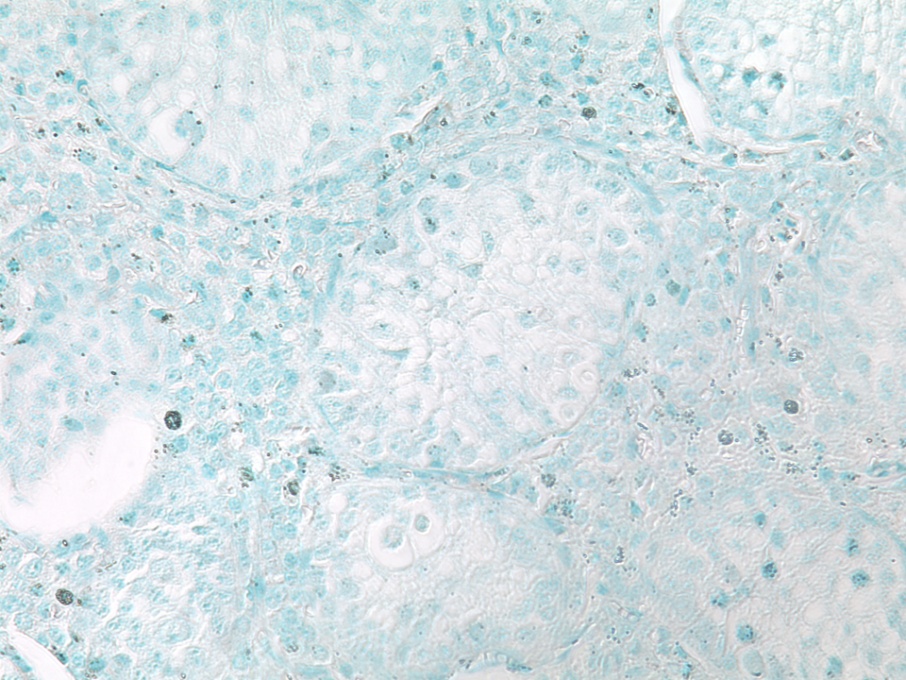


**Figure S4:** No significant apoptosis was present in chronically immunosuppressed, uninfected group
